# Correction: Feroz et al. Non-Muscle Myosin II A: Friend or Foe in Cancer? *Int. J. Mol. Sci.* 2024, *25*, 9435

**DOI:** 10.3390/ijms27187988

**Published:** 2026-09-08

**Authors:** Wasim Feroz, Briley SoYoung Park, Meghna Siripurapu, Nicole Ntim, Mary Kate Kilroy, Arwah Mohammad Ali Sheikh, Rosalin Mishra, Joan T. Garrett

**Affiliations:** 1 Department of Pharmaceutical Sciences, James L. Winkle College of Pharmacy, Cincinnati, OH 45229, USA; ferozwm@mail.uc.edu (W.F.); sarahpark0806@gmail.com (B.S.P.); meghna.siripurapu24@ihsd.us (M.S.); ntimne@mail.uc.edu (N.N.); kilroymk@mail.uc.edu (M.K.K.); rosalin.mishra@cchmc.org (R.M.); 2 Cancer Research Scholars Program, College of Allied Health Sciences, University of Cincinnati, Cincinnati, OH 45267, USA; 3 Division of Endocrinology, University of Cincinnati, Cincinnati, OH 45267, USA; sheikhaw@ucmail.uc.edu

In the original publication [1], the following ref. was not cited: 

Brito, C.; Sousa, S. Non-Muscle Myosin 2A (NM2A): Structure, Regulation and Function. *Cells* **2020**, *9*, 1590. https://doi.org/10.3390/cells9071590.

The citation has now been inserted in “Figure 5 Legend” and should read as follows:

**Figure 5.** The figure shows the structure and orientation of cell migration in the 2D environment. At the front, actin filaments within lamellipodia and filopodia are oriented with their rapidly polymerizing ends in the forward direction. In the main body, actin and myosin filaments form bipolar structures to aid in cell retraction. NM IIA and NM IIB show distinct localizations inside the cell, with NM IIA predominantly being found at the leading edge, where actin dynamics are most active. NM IIB is predominant toward the rear end. The region between the leading and trailing edges contains varying concentrations of NM IIA and NM IIB. Additional molecules, such as RhoA, Rac1, Cdc42, Ca^2+^ ions, and αPKC, also play significant roles in this cellular organization and migration process (Figure Adapted from [163]).

With this correction, the order of some references has been adjusted accordingly. The authors state that the scientific conclusions are unaffected. This correction was approved by the Academic Editor. The original publication has also been updated.
